# Robust, Self-Healing Superhydrophobic Fabrics Prepared by One-Step Coating of PDMS and Octadecylamine

**DOI:** 10.1038/srep27262

**Published:** 2016-06-06

**Authors:** Chao-Hua Xue, Xue Bai, Shun-Tian Jia

**Affiliations:** 1 College of Resource and Environment, Shaanxi University of Science and Technology, Xi’an 710021, China; 2 Shaanxi Research Institute of Agricultural Products Processing Technology, Shaanxi University of Science and Technology, Xi’ an 710021, China

## Abstract

A robust, self-healing superhydrophobic poly(ethylene terephthalate) (PET) fabric was fabricated by a convenient solution-dipping method using an easily available material system consisting of polydimethylsiloxane and octadecylamine (ODA). The surface roughness was formed by self-roughening of ODA coating on PET fibers without any lithography steps or adding any nanomaterials. The fabric coating was durable to withstand 120 cycles of laundry and 5000 cycles of abrasion without apparently changing the superhydrophobicity. More interestingly, the fabric can restore its super liquid-repellent property by 72 h at room temperature even after 20000 cycles of abrasion. Meanwhile, after being damaged chemically, the fabric can restore its superhydrophobicity automatically in 12 h at room temperature or by a short-time heating treatment. We envision that this simple but effective coating system may lead to the development of robust protective clothing for various applications.

Biomimetic superhydrophobic surfaces, as inspired by Lotus plant leaves and water striders, have attracted tremendous attraction not only because of its scientific background but also for wide range of technological applications including self-cleaning^1^,^2^,^3^,^4^,^5^,^6^,^7^ anti-fouling^8^,^9^, anti-corrosion^10^,^11^,^12^, oil-water separation^13^,^14^,^15^,^16^,^17^,^18^,^19^ and so forth. After decades of study, it has been well recognized that the combination of micro- and nanoscaled hierarchical structures and low surface energy materials is crucial to fabricate numerous artificial superhydrophobic surfaces^20^,^21^,^22^,^23^,^24^. In recent years, considerable efforts have been devoted to preparing superhydrophobic materials for various purposes. Nevertheless, it is still a major challenge to resolve the durability of the superhydrophobic surfaces for practical applications^25^,^26^,^27^.

Living organisms, which are well known as a self-healing function, can survive when even a considerable part is damaged or completely lost. Inspired by that, self-healing superhydrophobic surfaces were fabricated to extend the lifespan of these coatings, which is actually of importance for the employment of the superhydrophobic materials in practical applications^28^,^29^,^30^,^31^,^32^,^33^,^34^,^35^,^36^,^37^,^38^. Generally speaking, self-healing of the superhydrophobic surfaces can be realized by migrating of hydrophobic components^34^,^39^,^40^,^41^,^42^,^43^,^44^,^45^,^46^,^47^ or regenerating topographic structures^48^,^49^,^50^,^51^,^52^,^53^. For practical application, developing a simple and facile method of fabricating self-healing superhydrophobic surfaces becomes the urgent demand. Though several works have been reported recently^19^,^28^,^29^,^30^,^54^, further studies should be done in future to advance these materials into practice.

Octadecylamine (ODA) is well known as a low surface energy material and has been widely used to fabricate self-healing superhydrophobic surfaces based on the migration of ODA molecules^55^,^56^,^57^,^58^. Unexpectedly, when ODA was employed as a low energy material to prepare superhydrophobic surfaces, something interesting happens during our experiments. As the concentration of ODA reached a certain amount, rough surfaces were formed with hierarchical micro- and nanoscale sheet structures by assembly of ODA. Within this context, the construction of superhydrophobic surface is more effective due to without using any lithography steps or adding any nanomaterials to form roughness.

Herein, we report a new coating system that can make poly(ethylene terephthalate) (PET) fabrics with a durable self-healing superhydrophobicity using a one-step wet-chemistry coating technique. The coating system consists of two commonly used materials, namely, polydimethylsiloxane (PDMS) and ODA, as shown in Fig. 1. This simple and low cost coating possessed remarkable durability against different pH solutions and exhibited excellent resistance to repeated machine washes and several abrasion damages, whilst retaining its superhydrophobicity. Moreover, the coating showed remarkable self-healing ability against both chemical and physical damages.

## Methods

### Materials

Polydimethylsiloxane (PDMS, Sylgard 184 Silicone Elastomer Kit with components of PDMS base and curing agent) was purchased from Dow Corning. Octadecylamine (ODA) was purchased from Aladdin Industrial Co., Ltd. Tetrahydrofuran (THF) and distilled water was commercially obtained. Soap powder (Qiqiang KEON brand, Nafine Chemical Industry Group Co., Ltd., China) with sodium dodecyl benzene sulfonate as the main ingredient was purchased from a supermarket. All chemicals were used without further purification. PET fabric (plain weave, 16^s^ + 16^s^ × 10^s^, 84 × 35, 230 g/m^2^) was purchased from a local market, which is a polyester of poly(ethylene and terephthalate) (Fig. S1).

### Fabrication of Superhydrophobic PET Fabrics

A PDMS solution was prepared by dissolving 1 g of PDMS and 0.1 g of curing agent into 48.9 g of THF solution. The solution was ultrasonicated for 15 min to form the Solution A. ODA (2 g) was dissolved to THF (48 g) by heat to form the Solution B. Prior to coating treatment, the Solutions A and B were mixed together to form a coating solution. Then the PET fabric samples (10 cm × 10 cm) were immersed in the as-prepared coating solution for 3 min and finally dried at 40 °C for 30 min to obtain PDMS/ODA-coated PET fabric. For the sake of comparison, PDMS-coated PET fabric and ODA-coated PET fabric were also prepared with the same procedure, in which PDMS solution was prepared by dissolving 1 g of PDMS and 0.1 g of curing agent into 98.9 g of THF solution, and ODA solution was prepared by dissolving 2 g of ODA into 98 g of THF solution under heating.

### Characterization

Scanning electron microscopy (SEM) images were obtained on a Hitachi S-4800 field emission scanning electron microscope operated at an acceleration voltage of 3 kV. Samples were sputter-coated with gold prior to examination. The surface composition of coating was measured by X-ray photoelectron spectroscopy (XPS, Kratols Axis Supra) using Al Kα radiation at a 90 take-off angle. All the binding energy values were calibrated using the reference peak of C1_S_ at 284.6 eV. Spectra were evaluated using the Casa XPS software, version 2.3.15 from Casa Software Ltd. (United Kingdom). The symmetrical GL(30) line shape was applied, which consists of a Gaussian (70%) and a Lorentzian (30%) component. Water contact angles (CA) of the fabrics were measured with a deionized water droplet of 5 μL on a video optical contact angle system (OCA 20, Dataphysics, Germany) at room temperature, and images were captured with a digital camera. The reported values of CA and sliding angle (SA) were determined by averaging values measured at five different points on each sample surface.

### Stability Evaluation of Superhydrophobic PET Fabrics

The abrasion resistance of the superhydrophobic fabrics was tested according to a modified procedure based on the AATCCA Test Method 8–2001. The test was performed under a commercial abrasion tester (Y571L(A), Lai Zhou, China). In our experiment, the uncoated fabric was as the abrasion partner. The sample was fixed onto the stainless steel column and moved repeatedly with a load pressure of 45 kPa (200 mm for one cycle). After certain cycles of abrasion, the CA on the rubbed area of the sample was tested.

The washing durability of the superhydrophobic fabrics was tested by a standard procedure according to AATCC Test Method 61–2003 Test No. 1A. The fabric sample was washed using a laundering machine (SW-12 AII, Da Rong, China) at 40 °C with water containing 0.37 wt% soap powder and 10 stainless steel balls with diameter of 6 mm and weight of 0.9 g. After 45 min of laundering, the laundered sample was rinsed with abundant tap water to remove the residual detergent and dried at 40 °C without tension. The CA and SA were then measured. This standard washing procedure is equivalent to five cycles of home machine laundering. For convenience, we used equivalent number of home machine laundering in this work. And in order to investigate the effect of washing temperature on superhydrophobicity, we also conducted the above-mentioned washing process at 80 °C.

The chemical durability of the superhydrophobic fabrics was evaluated by immersing the samples into aqueous solutions of different pH values.

### Self-Healing Property Evaluation of Superhydrophobic PET Fabrics

The healing ability of the superhydrophobic fabrics was evaluated by air plasma etching of the substrates using an YZD08-5C plasma cleaner (Tangshan Yanzhao Science and Technology Institute, China) at high vacuum under a power of 19 W for 1 min, followed by storage at room temperature or heating for self-healing of the superhydrophobicity and CA measurement^30^,^31^,^32^. Such a plasma treatment can make fabrics hydrophilic with CA around 50°.

## Results

### Morphology of Fabrics

**Figure 2:**
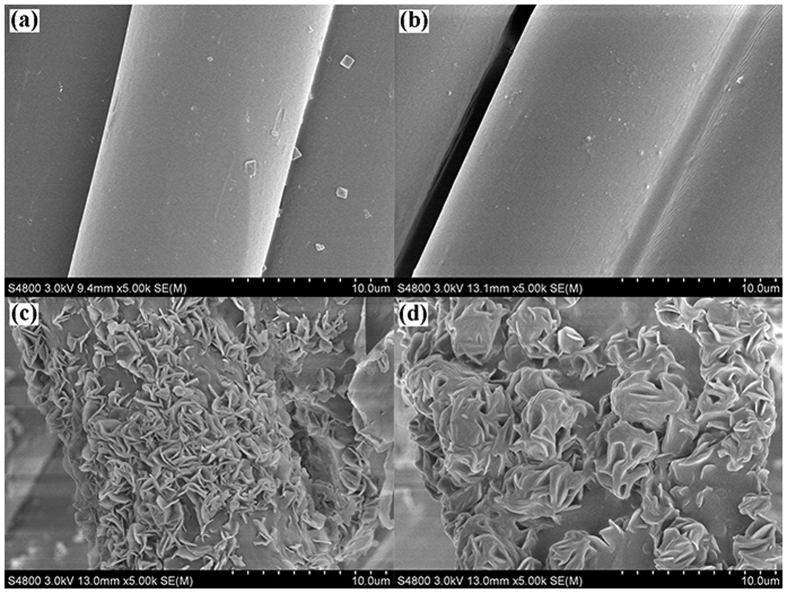
SEM images of (**a**) pristine PET fiber, (**b**) PDMS-coated PET fiber with a PDMS concentration of 1 wt%, (**c**) ODA-coated PET fiber with a ODA concentration of 2 wt%, (**d**) PDMS/ODA-coated PET fiber (PDMS 1 wt%, ODA 2 wt%).

SEM was employed to characterize the morphology of the surfaces on the PET fabrics. It was found that the pristine PET fiber showed a smooth surface (Fig. 2a). After coating with PDMS, the morphology of the PET fiber maintained almost unchanged (Fig. 2b). Interestingly, a sheet structure with hierarchical microscale and nanoscale roughness was formed when coating ODA on PET fibers, which is a key factor in obtaining superhydrophobicity, as shown in Fig. 2(c). Moreover, when coating the mixture of PDMS and ODA on PET fabrics, the hierarchical microscale and nanoscale structures can still maintained, which looks like flower clusters (Fig. 2d). The as-formed structure helps to complement the microscale roughness inherent in the fabric weave, directing to proper roughness for superhydrophobic surfaces.

### Superhydrophobic Property of Fabrics

**Figure 3:**
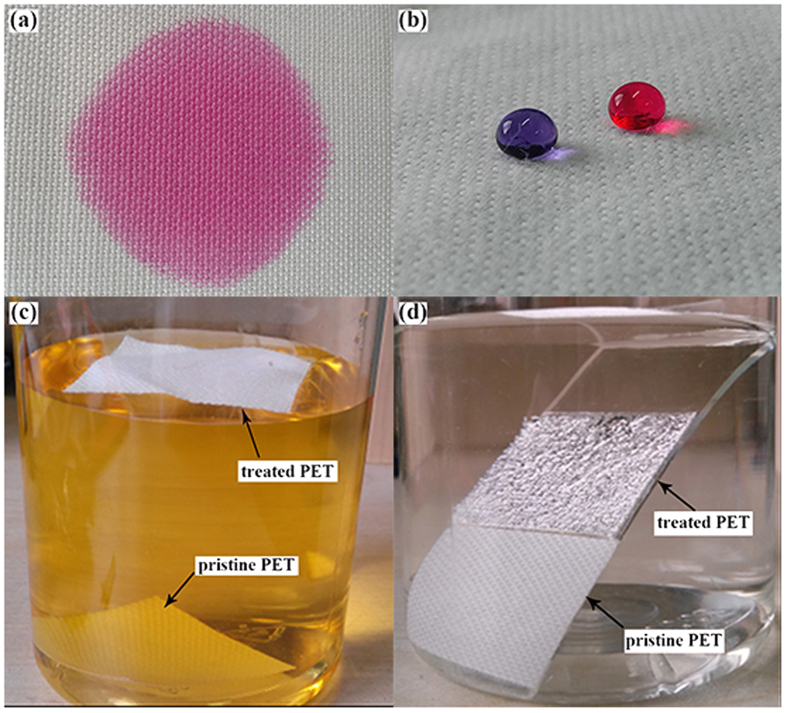
The pictures of water drops on (**a**) pristine and (**b**) PDMS/ODA-coated PET fabrics; (**c**) free immersion of pristine PET and PDMS/ODA-coated PET fabrics in dyed water, (**d**) immersion of pristine PET and PDMS/ODA-coated PET fabrics stuck on glass into water.

Surface wettability characterization showed that the water can spread easily on the pristine PET fabric due to the capillary effects of the fibrous structure, as shown in Fig. 3(a). After coating with PDMS and ODA respectively, the CA of the PDMS-coated fabric increased to 156.6° ± 2.1° with SA of 15° ± 3°, and the CA of the ODA-coated fabric is 160.2° ± 2.5° with SA of 6° ± 1°. In comparison, it seems that PDMS/ODA-coated PET fabric has similar superhydrophobicity with CA of 161.3° ± 3° and SA of 4.5° ± 1.6°. However, the CA of the PDMS/ODA-coated PET fabric was obviously higher than that of the fabrics treated by single-component of PDMS or ODA with SA decreased, indicating that the hierarchical microscale and nanoscale roughness caused by ODA can obviously increase the superhydrophobicity of the fabric. As shown in Fig. 3(b), the fabric coated with PDMS/ODA showed remarkable superhydrophobicity. To further prove the excellent water repellency, the coated fabric was completely immersed into water, and then the wettability was observed. Obviously, the pristine PET fabric sunk under water, whereas the coated fabric floated on the surface of water after release of force (Fig. 3c). In order to show visually the air pocket formation on the superhydrophobic fabric, the fabric were stuck on a glass slide and then immersed into water. As expected, the coated fabric shows an obvious bright plastron layer due to the total reflectance of light at the air layer trapped on the surface (Fig. 3d). This trapped air can effectively prevent the fabric from wetting by water. While under the same condition, the pristine PET fabric showed no bright plastron layer. This phenomenon indicated that the coated fabrics possessed typical Cassie^59^ mode superhydrophobicity.

### Durability of the Superhydrophobic Fabrics

**Figure 4:**
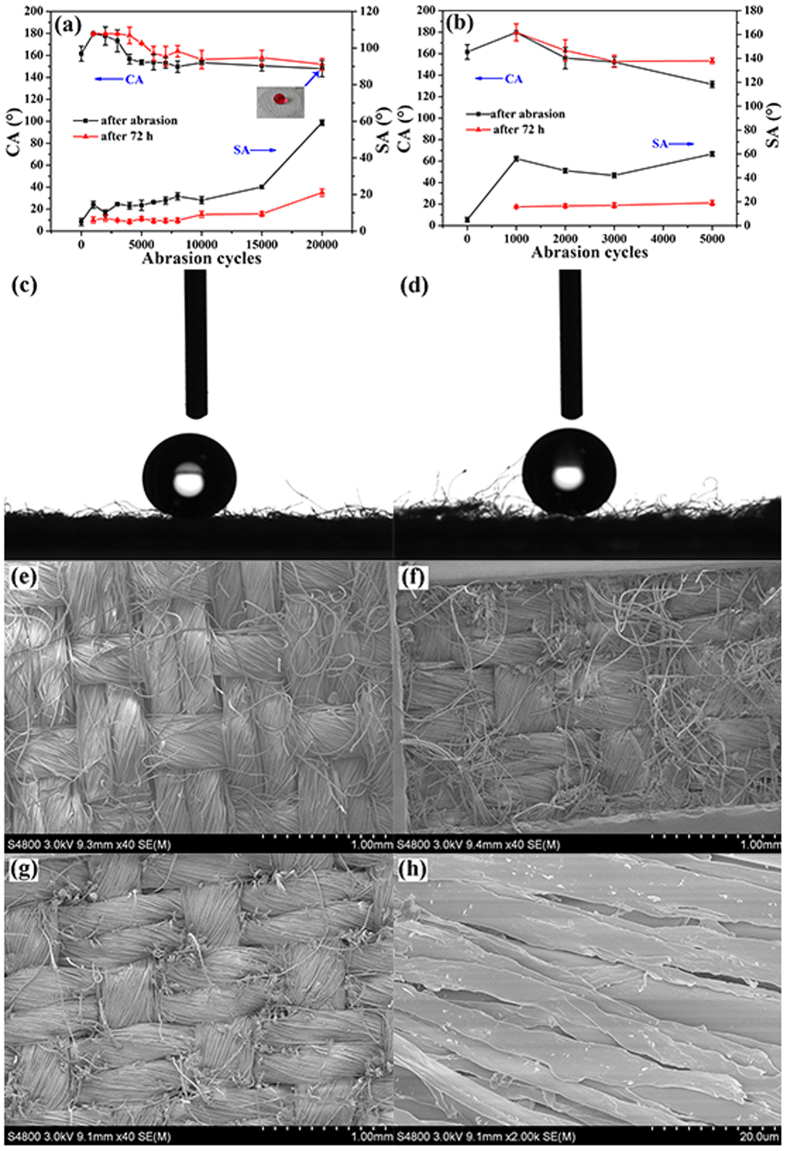
CA and SA change of (**a**) PDMS/ODA-coated PET fabric, (**b**) ODA-coated PET fabric with abrasion cycles; CA of the (**c**) PDMS/ODA-coated PET fabric and (**d**) PDMS/ODA-coated PET fabric after abrasion test of 1000 cycles. (**e**) SEM image of pristine PET fabric. (**f**) SEM images of the PDMS/ODA-coated PET fabric after abrasion test of 1000 cycles, (**g**) 20000 cycles, (**h**) is the high magnification image of (**g**).

In practical application, the durability of the superhydrophobic fabric is a critical issue. Abrasion and laundering durability of the coated fabrics were then evaluated. For fabrics in everyday routine uses, the physical damages occur more likely from gentle abrasion, such as when coming into contact with other fabrics. The abrasion durability of the coated fabric was tested using the modified procedure based on the AATCCA Test Method 8–2001 with an untreated fabric to simulate actual wear. Fig. 4(a,b) shows the effect of abrasion cycles on the CA and SA of the coated fabric. After 1000 cycles of abrasion, the PDMS/ODA-coated fabric showed an obviously improved superhydrophobicity with the CA increasing from 161.6° ± 3° to 179.8° ± 0.1° (Fig. 4c,d). From Fig. 4(d), it can also be clearly observed that an air pocket existed between the fabric and water drop. Such a trend is completely different to the results reported by our previous papers, in which the liquid repellency reduces monotonously with increasing the abrasion cycles^2^,^8^,^14^,^18^,^25^. It suggests that the liquid resistant performance of the superhydrophobic fabrics could be increased during the initial practical use. The possible reason for this phenomenon is that some protruding fuzzes induced by mechanical abrasion, which can act as a scaffold to support the water, as shown in Fig. 4(e,f). However, the SA of the fabric increased a lot in the first 1000 cycles of abrasion. This might be due to the appearance of protruding fuzzes, which caused some elastic force to the water on the fabric. Thus, the SA increased with increasing the abrasion cycles. Meanwhile, when extending the cycles of abrasion to 2000, the SA of the fabric decreased a little. This might due to the disappearance of some protruding fuzzes by severe mechanical friction. After that, the SA of the fabric increased with increasing the abrasion cycles due to loss of surface roughness and coating. Although the CA decreased with further increasing the abrasion cycles, the coated fabric can withstand at least 5000 cycles of abrasion damage without losing its superhydrophobicity. Nevertheless, after 20000 cycles of abrasion under the pressure of 45 kPa, the CA of the coated fabric declined to less than 150° and the SA increased to 59.3° with an error of  ± 0.7°. SEM characterization (Fig. 4g) clearly revealed that nearly no protruding fuzzes could be observed from the top surface of the fabric. To further observe the morphology of fibers, the high magnification SEM image was taken. It can be found that some fibers were abraded broken after 20000 abrasion cycles (Fig. 4h). The reduced liquid repellency after abrasion was mainly attributed to the partial loss of surface roughness and the removal of PDMS/ODA at the fiber surface. Interestingly, when the worn fabric was stored at room temperature for 72 h, it could restore its superhydrophocicity with CA of 152° ± 2° and SA of 21° ± 1°. This might be caused by the migration of PDMS or ODA from the interstices of the PET fibers. And the results clearly demonstrated that the worn PDMS/ODA-coated PET fabric is still capable of healing its superhydrophobicity. By contrast, ODA-coated PET fabric has the similar phenomenon with PDMS/ODA-coated fabric upon abrasion. However, when the abrasion cycles increased to 5000, the ODA-coated PET fabric lost its superhydrophobicity with CA of 131.5° ± 1.5° (Fig. 4b). Compared with PDMS/ODA mixtures, the structure of ODA on PET fibers is easier to be leveled because ODAs are small molecules having weaker interaction among molecules or between ODA molecules and PET fiber surfaces. Therefore, the SA of the abraded ODA-coated PET fabric after recovery reached 20°, which is much higher than that of the PDMS/ODA-coated PET fabric within 5000 abrasion cycles. Thus, we concluded that the remarkable abrasion resistance of PDMS/ODA-coated PET fabric mainly comes from strong adherence of PDMS to fibers, because PDMS is an elastic silicone rubber and possessed excellent strength to wearing^9^,^18^.

**Figure 5:**
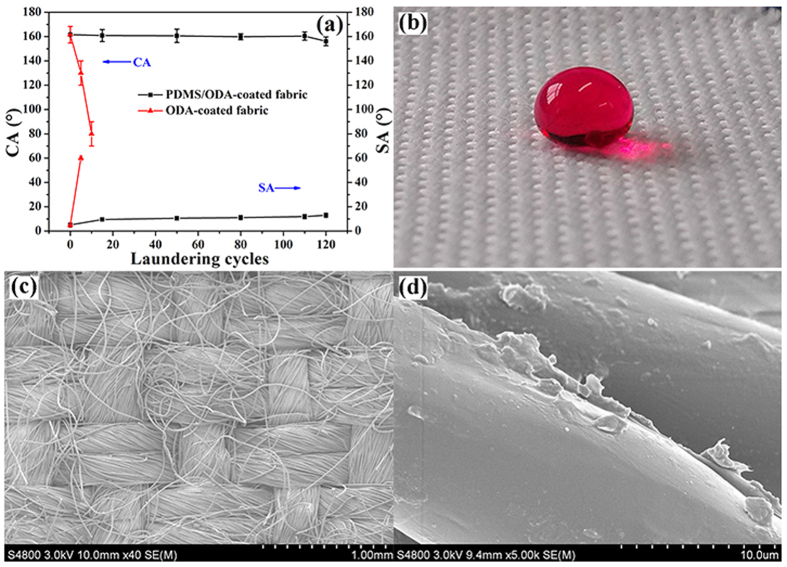
(**a**) CA and SA change of coated PET fabric with laundering cycles, (**b**) photograph of a water drop on the PDMS/ODA-coated PET fabric after laundering test of 120 cycles, (**c**) SEM images of PDMS/ODA-coated PET fabric after laundering test of 120 cycles, and (**d)** is the high magnification image of (**c**).

Laundering durability of the coated fabric was evaluated by reference of a standard machine laundry process according to AATCC Test Method 61–2003 Test No. 1A. Figure 5(a) shows the change in the value of CA and SA with laundering cycles. The CA of the ODA-coated fabric decreased sharply to 80° ± 5° when the samples were laundered to 10 cycles. Contrarily, the PDMS/ODA-coated fabric can withstand at least 120 cycles of home laundering without losing its superhydrophobicity, as shown in Fig. 5(b). With increasing laundering cycles, the CA of the PDMS/ODA-coated fabric slightly decreased and the SA increased a little. This phenomenon indicates that a partial removal of PDMS or ODA occurs during the laundering process (Fig. 5c,d). Whereas, both changes in CA and SA were less than 8°, demonstrating that the PDMS/ODA-coated fabric possessed excellent durability of the superhydrophobicity to washing. Considering that higher temperature washing might occur in application, laundering cycles at 80 °C were also conducted. It was found that the superhydrophobicity of the fabric after washing at 80 °C decreased a little faster with washing cycles than that at 40 °C, however the fabrics still sustained the superhydrophobicity with CA over 150° after 80 washing cycles (See Fig. S2).

**Figure 6:**
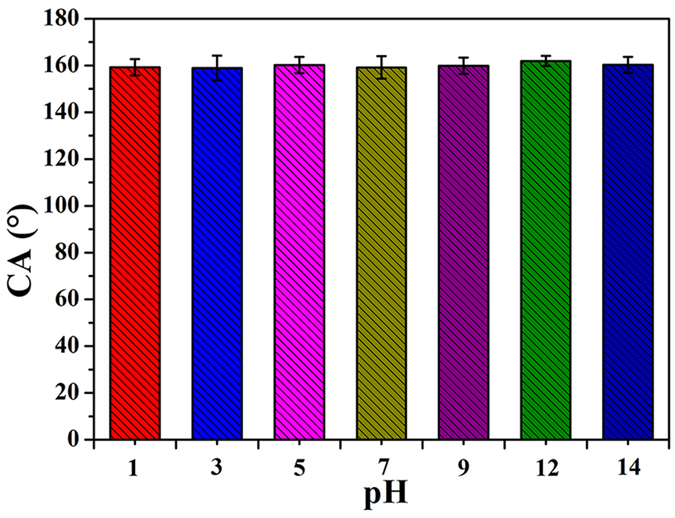
CAs of the PDMS/ODA-coated PET fabric with different pH solutions.

Apart from abrasion and laundering test, we also evaluated the durability of superhydrophobicity of the PDMS/ODA-coated fabric by immersing the samples into solutions with different pH (pH = 1–14) for 24 h. Figure 6 presents that the CAs of the sample were nearly unchanged, indicating their strong resistance to different pH solutions.

### Self-healing of Superhydrophobicity of PDMS/ODA-Coated PET Fabrics

**Figure 7:**
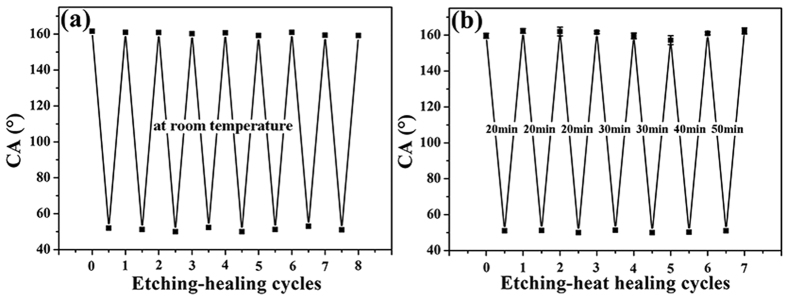
Self-healing cycles of PDMS/ODA-coated PET fabric (**a**) automatically healed at room temperature, (**b**) healed by heat.

PDMS/ODA-coated PET fabrics were damaged with air plasma to investigate their self-healing ability at room temperature. This process decomposes the outermost PDMS or ODA on the surface of the coated fabric, as sunlight can do when the superhydrophobic PET fabric is for outdoor applications. It was found that air plasma treatment under a power of 19 W for 1 min could turn the superhydrophobic PET fabrics into hydrophilic ones with CA around 50°. This hydrophilic transformation indicated that the PDMS/ODA surface layer was partially decomposed by the chemical reaction with highly reactive oxygen radicals and oxygen ions and introduction of polar groups. Nevertheless, after exposing the air-plasma-etched PET fabric in room temperature for 12 h, the original superhydrophobicity was restored, demonstrating that the surface of the damaged superhydrophobic coating was covered again with the hydrophobic PDMS/ODA. As shown in Fig. 7(a), the etching-healing process can be repeated at least 8 times without decreasing the superhydrophobicity, showing that the PDMS/ODA-treated PET fabrics have a strong ability to heal the damaged superhydrophobicity. In our experiment, we also found that the self-healing ability of the superhydrophobic fabrics is temperature-dependent, with a more accelerated self-healing process under a higher temperature of 40 °C. As described in Fig. 7(b), when the PDMS/ODA-coated PET fabric treated by plasma was heated at 40 °C for 20 min, its superhydrophobicity was restored in the first three cycles. However, with the increasing of etching cycles, the time needed to recover the superhydrophobicity of the coatings became longer, meaning that the healing became more difficult. This may be due to the rotation and movement of the PDMS/ODA chains that are thermodynamically driven by minimizing the surface tension^33^,^34^,^39^. To explain this phenomenon, we propose the self-healing process of the superhydrophobic coating as follows. Once the outermost layer of the PDMS/ODA on the fabric is decomposed by the plasma treatment, the surface of the coated fabric becomes hydrophilic, which drives the underlying hydrophobic chains of PDMS/ODA migrate to the coating surface to lower its surface energy. As a result, the introduced polar groups tend to be hidden inside the coating layer, and more PDMS/ODA chains were exposed to the surface. Thus, the damaged superhydrophobicity of the fabric is repaired. However, with increasing etching-healing cycles, some of the PDMS/ODA of the coating is consumed. In this case, the rotation and movement of PDMS/ODA chains becomes more difficult and the healing process becomes slower^31^,^32^,^33^,^34^.

**Figure 8:**
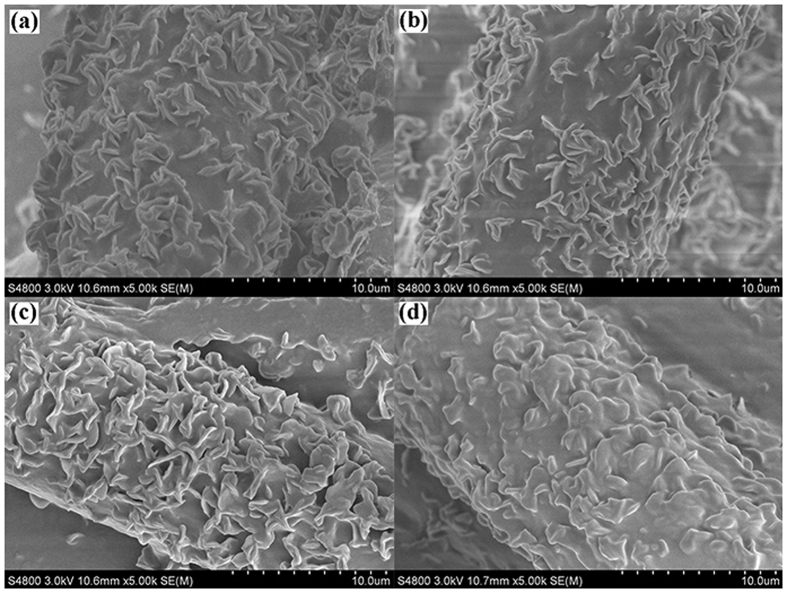
SEM images of PDMS/ODA-coated PET fabric (**a**) after 1^st^ plasma treatment, (**b**) after 1^st^ automatically healed at room temperature for 12 h, (**c**) after 8 times etching-healing cycles at room temperature, (**d**) after 7 times etching-heating cycles.

SEM image confirms that the fabrics after several etching-healing cycles still have micro- and nanoscaled hierarchical structures that are essential for superhydrophobicity (Fig. 8c), whereas, the structure of the healed coating is a little different from the original ones. From Fig. 8(a,b), the morphology of the coating being treated by air plasma and healed at room temperature for 12 h is almost unchanged. Thus, the change of coating structure may be caused by structure rearrangement of PDMS/ODA during the etching process. Therefore, we concluded that the self-healing ability of the coating is caused by the migration of PDMS/ODA chains. Similarly, the coated fabric after 7 times air-plasma-heat-treatment still maintained the micro- and nano-scale hierarchical structure. However, the fiber surface became a little different compared with the fabric before (Fig. 8d). This may be caused by the partially melt of ODA during the heat process due to its low fusion point (50–54 °C)^56^.

**Figure 9:**
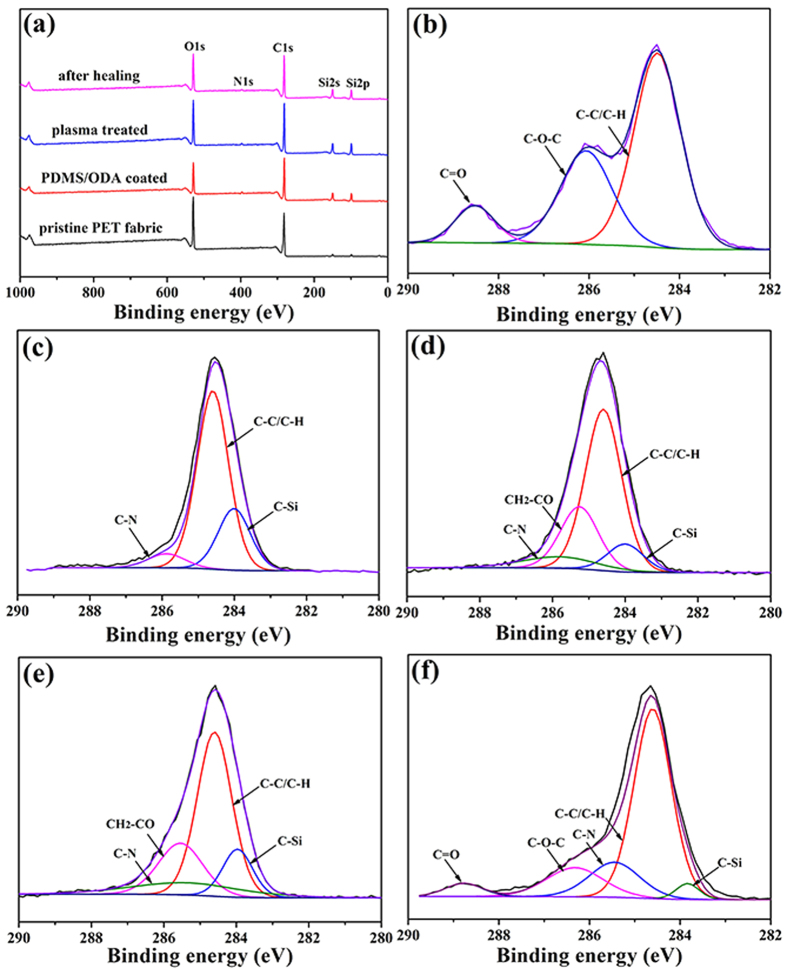
(**a)** XPS survey spectra of the PET fabric before and after various treatments; C1s XPS spectra with fitting curves of (**b**) pristine PET, (**c**) PDMS/ODA-coated PET fabric; PDMS/ODA-coated PET fabric (**d**) after air plasma treatment, (**e**) after healing at room temperature, (**f**) after 20000 cycles of abrasion and healing.

The chemical composition of the coating was analyzed by XPS (Fig. 9). As shown in Fig. 9(a), the survey spectrum indicates that the pristine PET fabric was composed of elements C and O. After coating with PDMS/ODA, the new Si and N signals were detected, indicating that PDMS and ODA have been incorporated onto the surface of the PET fibers. To further prove this, high-resolution XPS C1s spectra of the fabric before and after coating treatment were analyzed. Figure 9(b) shows that the C1s core-level spectra of pristine PET fabric can be curve-fitted into peaks with binding energies at 288.5, 286.1 and 284.6 eV, which are the typical characteristics of C=O, C-O-C and C-C/C-H moieties, respectively. And Fig. 9(c) corresponds to the C1s core-level spectra of PDMS/ODA-coated PET fabric. After coated with PDMS and ODA, the peaks of C-N (285.8 eV) and C-Si (283.8 eV) can be curve-fitted from the spectra. The surface chemical composition data (Table S1) of these surfaces measured by XPS show that the O/C concentration ratio of PDMS/ODA-coated PET fabric is 0.25, while after 1 min plasma treatment it rises to 0.32, indicating that polar groups containing O were induced at the surface. However, the O/C concentration ratio returned to 0.27 after self-healing, indicating that non-polar chains or molecules of PDMS/ODA reversed to the outermost layers of the coatings, realizing the recovery of low surface energy. In order to further investigate the self-healing mechanism of the fabric, the high-resolution XPS C1s spectrum of the etched fabric was analyzed. It was found that a new curve-fitted peak indeed appeared at 285.2 eV, which was ascribed to the CH_2_-CO moiety and this might be caused by the decomposition of ODA (Fig. 9d). After healing, the 285.2 eV peak still existed, while the intensity was weakened (Fig. 9e). In contrast, there is no other new peaks detected in the fabric after 20000 cycles of abrasion and healing, except for the appearance of the peaks of C=O and C-O-C (Fig. 9f) which are from the PET polymers of the fibers, showing the revealing of PET fibers due to the peeling off of the coating after severe abrasion. These demonstrated that plasma treatment could cause chemical changes of the coating resulting in hydrophilic property and storage of the damaged fabrics at room temperature or heating could self-heal the superhydrophobicity, while abrasion cannot change the chemical composition of the coating.

## Conclusion

A robust, self-healing superhydrophobic PET fabric can be prepared by a one-step coating technique using an easily available materials system consisting of PDMS and ODA. The self-roughed property of ODA provides enough roughness for the fabric to achieve superhydrophobicity. It was shown that the as-prepared fabrics possessed remarkable durability to abrasion, washing, and different pH solutions. Moreover, the resultant fabric could automatically and repeatedly heal their superhydrophobicity after air-plasma-treatment at room temperature or heating, showing excellent resistance to chemical oxidation or strong light. This unique self-healing ability can significantly prolong the lifespan of superhydrophobic PET fabric in outdoor applications. The present fabrication method of robust, self-healing superhydrophobic PET fabric is simple and requires no special equipment, which is suitable for large-scale production.

## Additional Information

**How to cite this article**: Xue, C.-H. *et al*. Robust, Self-Healing Superhydrophobic Fabrics Prepared by One-Step Coating of PDMS and Octadecylamine. *Sci. Rep.* **6**, 27262; doi: 10.1038/srep27262 (2016).

## Supplementary Material

Supplementary Information

## Figures and Tables

**Figure 1:**
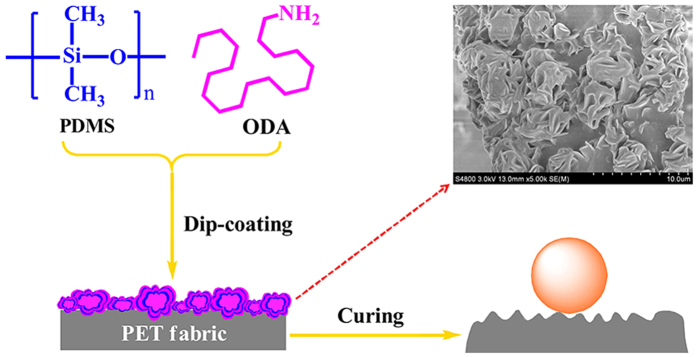
Schematic illustration of the preparation procedure for superhydrophobic fabrics.
