# Supplementary material for: Robust, Self-Healing Superhydrophobic Fabrics Prepared by One-Step Coating of PDMS and Octadecylamine
Source: Sci Rep. 2016 Jun 6;6:27262. doi: 10.1038/srep27262 (PMC4893697; doi:10.1038/srep27262)
Supplement: Supplementary Information [file srep27262-s1.doc]

**Supplementary Information**

**Robust, Self-Healing Superhydrophobic Fabrics Prepared by One-Step Coating of PDMS and Octadecylamine**

**Chao-Hua Xue^1,2^, Xue Bai^1^, Shun-Tian Jia^1^**

^1^ College of Resource and Environment, Shaanxi University of Science and Technology, Xi’an 710021, China

^2^ Shaanxi Research Institute of Agricultural Products Processing Technology, Shaanxi University of Science and Technology, Xi’ an 710021, China

Correspondence and requests for materials should be addressed to C.H.X. (email: [xuech@zju.edu.cn](mailto:xuech@zju.edu.cn))


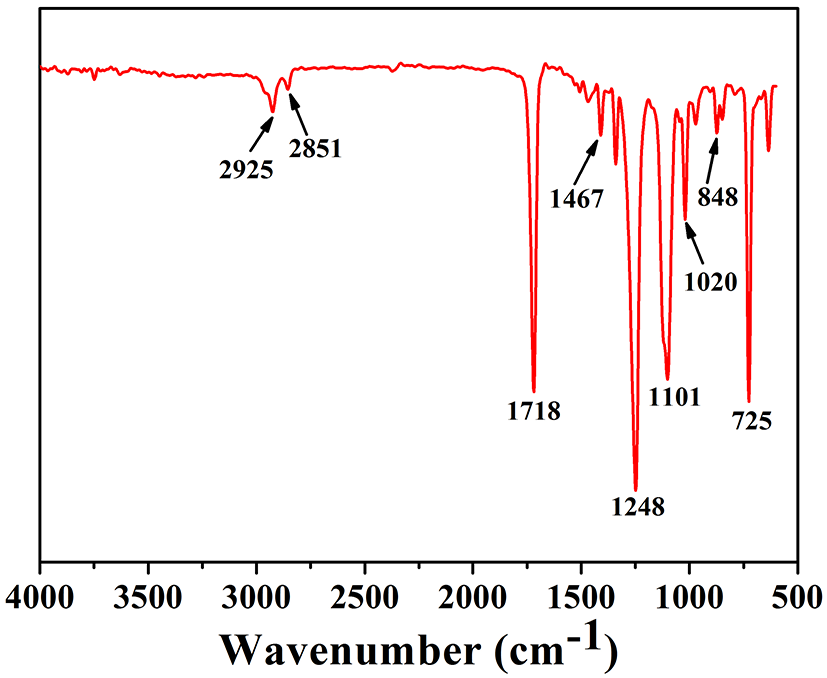


Figure S1 FT-IR of PET

The figure above showed the shark bands at 2925 and 2851 cm^-1^ arising from C-H stretching vibration of PET. The peak at 1718 cm^-1^ corresponded to the carbonyl stretching absorption, while the peak observed at 1467 cm^-1^ represented the benzene skeleton vibration absorption. The absorption signal observed at 1248 and 1101 cm^-1^ corresponded to the C-O-C stretching vibration of ester group. Meanwhile, the peaks which appeared at 848 and 1020 cm^-1^ were attributed to the C-H vibration of benzene and =C-O vibration, respectively. And the peak at 725 cm^-1^ is the C-H (CH_2_) out-of plane bending vibration of benzene. All the above characteristic peaks confirmed qualitatively that the fabric is a kind of PET.^1-3^


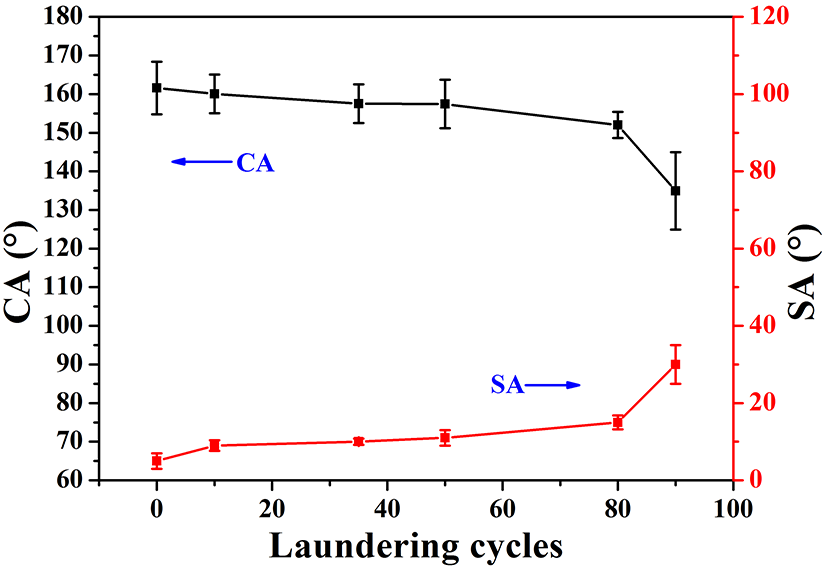


Figure S2 (a) CA and SA change of PDMS/ODA-coated PET fabric with laundering cycles (80℃).

Table S1. Surface chemical composition of PDMS/ODA-coated superhydrophobic coating determined using XPS analysis.

| Samples | Surface concentration (atom %) | | | | O/C |
| --- | --- | --- | --- | --- | --- |
|  | C | N | O | Si |  |
| PDMS/ODA-coated fabric | 66.22 | 1.39 | 16.71 | 15.68 | 0.25 |
| plasma-treated fabric | 63.26 | 1.44 | 20.33 | 14.97 | 0.32 |
| Self-healing 12h | 63.96 | 1.37 | 17.38 | 17.29 | 0.27 |

**Reference**

1 Zhou, H., Wang, H., Niu, H., Gestos, A. & Lin, T. Robust, self-healing superamphiphobic fabrics prepared by two-step coating of fluoro-containing polymer, fluoroalkyl silane, and modified silica nanoparticles. *Adv. Funct. Mater.* **23**, 1664-1670 (2013).

2 Wang, H. *et al.* Durable, Self-Healing Superhydrophobic and Superoleophobic Surfaces from Fluorinated-Decyl Polyhedral Oligomeric Silsesquioxane and Hydrolyzed Fluorinated Alkyl Silane. *Angew. Chem., Int. Ed.* **50**, 11433-11436 (2011).

3 Li, M., Deng, T., Liu, S., Zhang, F. & Zhang, G. Superhydrophilic surface modification of fabric via coating with nano-TiO_2_ by UV and alkaline treatment. *Appl. Surf. Sci.* **297**, 147-152 (2014).
